# Supplementary material for: LncRNA JHDM1D-AS1 Is a Key Biomarker for Progression and Modulation of Gemcitabine Sensitivity in Bladder Cancer Cells
Source: Molecules. 2023 Mar 6;28(5):2412. doi: 10.3390/molecules28052412 (PMC10005151; doi:10.3390/molecules28052412)
Supplement: Supplementary file 1 [file molecules-28-02412-s001.zip › molecules-2230454-supplementary.pdf]

Supplementary Material

LncRNA *JHDM1D-AS1* Is a Key Biomarker for Progression and Modulation of Gemcitabine Sensitivity in Bladder Cancer Cells

Isadora Oliveira Ansaloni Pereira [1,†], Glenda Nicioli da Silva [1,*,†], Tamires Cunha Almeida [2], Ana Paula Braga Lima [1], André Luiz Ventura Sávio [3,4], Katia Ramos Moreira Leite [5] and Daisy Maria Fávero Salvadori [6]

1 Departamento de Análises Clínicas, Pharmacy School, UFOP—Federal University of Ouro Preto, Ouro Preto 35400-000, MG, Brazil
2 Laboratory of Pain and Signaling, Butantan Institute, Sao Paulo 05503-900, SP, Brazil
3 Departamento de Odontologia, Faculdade do Centro Oeste Paulista—FACOP, Piratininga 17490-000, SP, Brazil
4 Departamento de Ciências Médicas, Universidade do Oeste Paulista—UNOESTE, Jaú 19050-900, SP, Brazil
5 Departamento de Cirurgia, Medical School, USP—University of São Paulo, São Paulo 05508-060, SP, Brazil
6 Departamento de Patologia, Medical School, UNESP—São Paulo State University, Botucatu 01151-000, SP, Brazil
* Correspondence: nicioli@ufop.edu.br
† These authors contributed equally to this work.

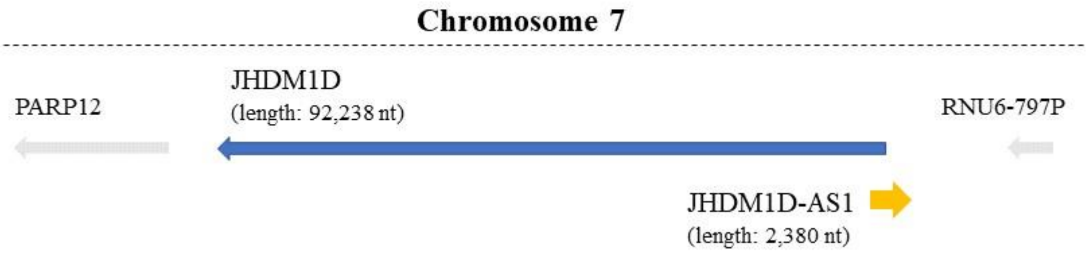

Figure S1. Chromosome location, structural organization, and length of *JHDM1D* and *JHDM1D-AS1* genes.

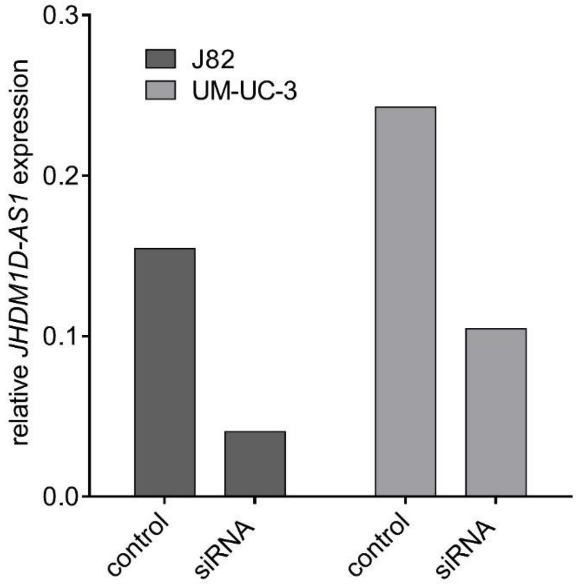

Figure S2. Relative expression of *JHDM1D-AS1* in J82 and UM-UC-3 cells after 72h of siRNA-*JHDM1D-AS1*.

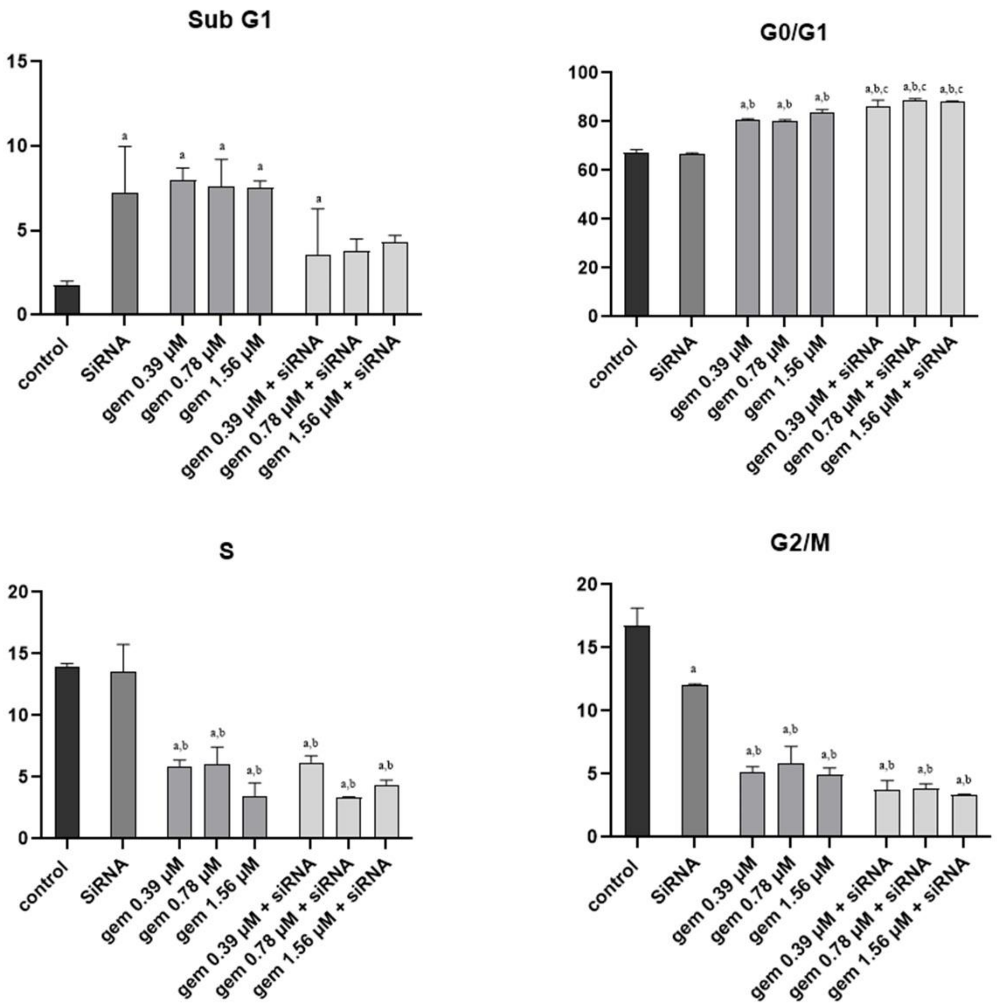

Figure S3. Percentage of J82 cells in each phase of cell cycle after treatment with gemcitabine, siRNA *JHDM1D-AS1*, or gemcitabine combined with siRNA *JHDM1D-AS1*. Control: untreated cells; gem: gemcitabine. a: $p < 0.05$ compared to control; b: $p < 0.05$ compared to siRNA; c: $p < 0.05$ compared to respective gemcitabine concentration alone.

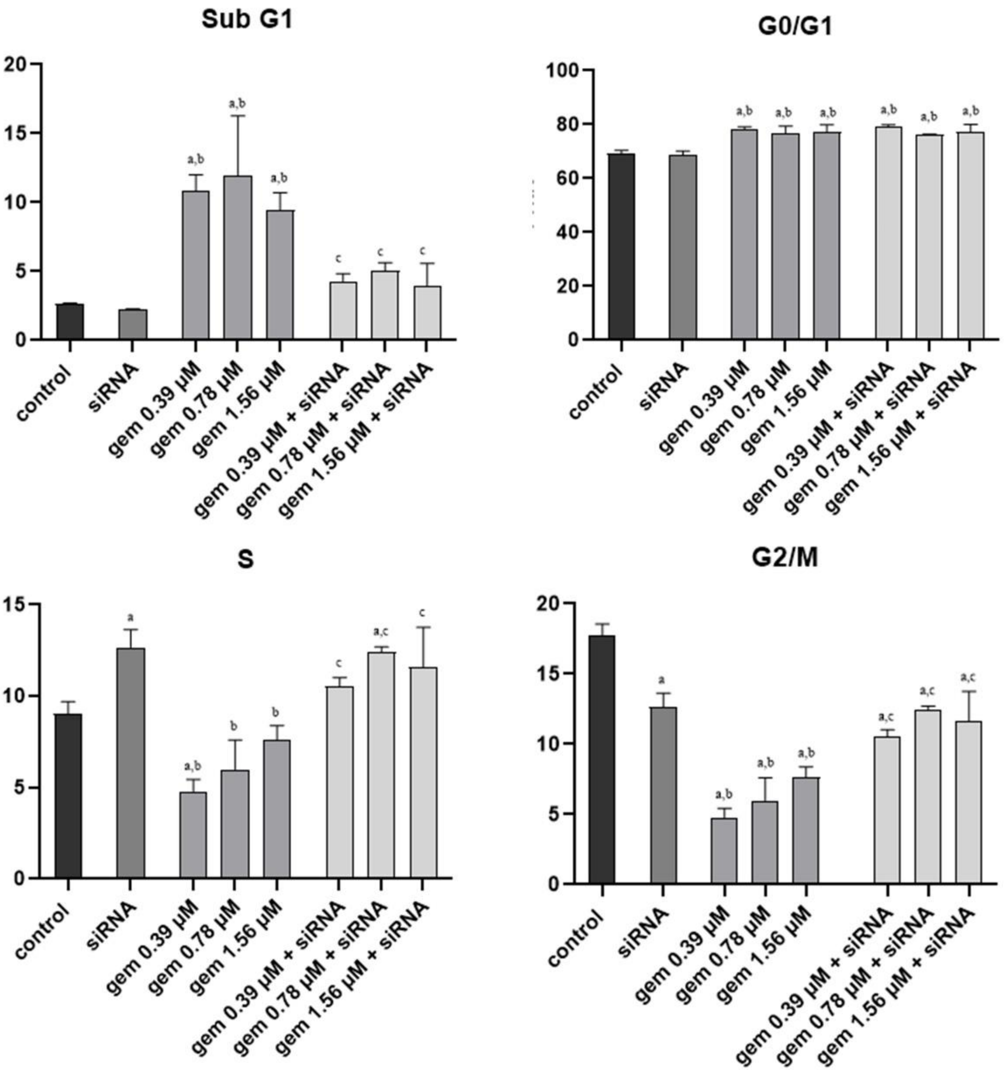

Figure S4. Percentage of UM-UC-3 cells in each phase of cell cycle after treatment with gemcitabine, siRNA *JHDM1D-AS1*, or gemcitabine combined with siRNA *JHDM1D-AS1*. Control: untreated cells; gem: gemcitabine. a: $p < 0.05$ compared to control; b: $p < 0.05$ compared to siRNA; c: $p < 0.05$ compared to respective gemcitabine concentration alone.

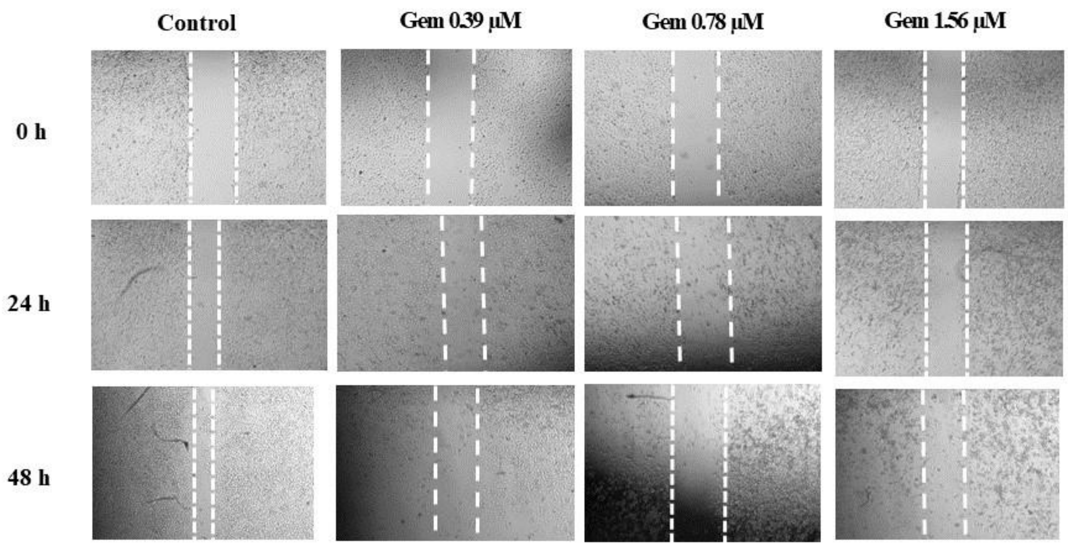

Figure S5. Photographs of cell migration in J82 cells after treatment with gemcitabine. Control: untreated cells; Gem: gemcitabine.

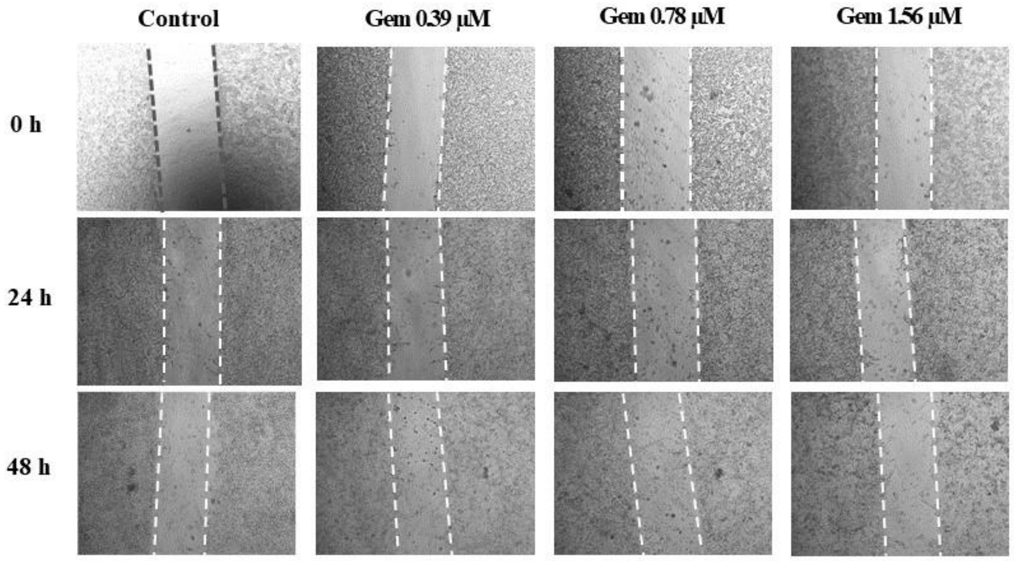

Figure S6. Photographs of cell migration in J82 cells after treatment with gemcitabine. Control: untreated cells; Gem: gemcitabine.
